# Analytical performance of thrombospondin-1 and cathepsin D immunoassays part of a novel CE-IVD marked test as an aid in the diagnosis of prostate cancer

**DOI:** 10.1371/journal.pone.0233442

**Published:** 2020-05-18

**Authors:** Annalisa Macagno, Alcibiade Athanasiou, Anja Wittig, Ramy Huber, Stephan Weber, Thomas Keller, Martin Rhiel, Bruno Golding, Ralph Schiess

**Affiliations:** 1 Proteomedix, Schlieren, Switzerland; 2 ACOMED Statistik, Leipzig, Germany; University of Minnesota Twin Cities, UNITED STATES

## Abstract

The Prostate Specific Antigen (PSA) test suffers from low specificity for the diagnosis of Prostate Cancer (PCa). We originally discovered two cancer-related proteins thrombospondin-1 (THBS1) and cathepsin D (CTSD) using a mass-spectrometry-based proteomics approach. The two serum proteins were shown to improve the diagnosis of high-grade PCa. Thus, we developed quantitative ELISAs for the determination of their concentration in human serum. Here we report their analytical performance in terms of limit of detection, specificity, precision, linearity and interferences, which were determined based on CLSI guidelines. Further, we investigated the influence of pre-analytical factors on concentration measurements. For this, blood from 4–6 donors was collected in different tubes and stored at room temperature for different times prior to centrifugation at different centrifugal forces and temperatures. Stability of THBS1 and CTSD under different storage temperatures was also evaluated. Our results show that the assays are specific, linear and sensitive enough to allow measurement of clinical samples. Precision in terms of repeatability and total within-laboratory coefficient of variation (CV) are 5.5% and 8.1% for THBS1 and 4.3% and 7.2% for CTSD, respectively. Relative laboratory-to-laboratory differences were -6.3% for THBS1 and -3% for CTSD. Both THBS1 and CTSD were stable in serum samples, with 80–120% recoveries of concentrations across donors, sample preparation and storage. In conclusion, the ELISAs as part of the novel commercial in vitro diagnostic test Proclarix are suitable for the use in clinical practice. THBS1 and CTSD can be accurately measured for their intended use independent of the lot and laboratory when conditions consistent with routine practice for PSA sampling and storage are used.

## Introduction

Prostate specific antigen (PSA) testing for the diagnosis of prostate cancer (PCa) results in a high false-positive rate, mostly due to the detection of elevated PSA levels in the blood when there is a benign disease such as enlargement or inflammation of the prostate [1]. This leads to many negative prostate biopsies. A genetics-guided discovery approach focusing on the PI3K/PTEN cancer pathway identified a panel of serum biomarkers improving diagnosis by distinguishing benign prostatic disease (BPH) from PCa [2]. From this panel, two glycoproteins, thrombospondin-1 (THBS1) and cathepsin D (CTSD), were shown to greatly improve the diagnosis when combined with free/total PSA ratio (%fPSA), particularly for patients in a diagnostic grey zone defined by total PSA of 2.0–10 ng/ml, negative digital rectal examination (DRE) and enlarged prostate volume (≥35 ml) [3, 4]. Namely, at 10% false-negative rate (90% sensitivity), 62% of cancer negative biopsies could be avoided in this group of patients, compared to 24% avoided if %fPSA was used alone [4].

THBS1 is an anti-angiogenic factor associated with several tumor types, including PCa, melanoma, breast, lung and bladder cancer [5–11]. Playing a role in tumor angiogenesis, it negatively correlates with PCa development [12–17]. Interestingly, THBS1 levels are lower in the serum of cancer patients underlining its anti-angiogenic role [18], a finding that applies also to PCa [3, 8]. CTSD is an aspartic endoprotease overexpressed and secreted by several tumor cell types, known to be associated with tumor aggressiveness and to be involved in PCa development promoting malignancy of prostatic epithelium [19–22]. Based on these data and our promising results highlighting the value of THBS1 and CTSD in improving PCa diagnosis, and given that no IVD assays for the determination of THBS1 and CTSD were available, we developed a commercial in vitro diagnostic (IVD) test (named Proclarix) that can be performed by any diagnostic laboratory. To be compatible with the routine measurements of PSA, which are commonly performed in serum, the test comprises two Enzyme-linked Immunosorbent Assays (ELISA) for the quantitative determination of THBS1 and CTSD in human serum. Proclarix is CE-IVD marked and contains the two ELISAs and a web-based software that integrates the concentrations of these biomarkers with tPSA, fPSA and age to calculate a risk score that can be used as an aid in the detection of high-grade PCa [23, 24]. Validation of Proclarix on 955 patients from two reference centers at resulted in a sensitivity of 90% for significant PCa (Gleason score ≥7, in biopsy specimen), a specificity of 43% and an NPV of 95% in comparison to a specificity of 17% and NPV of 89% for %fPSA alone. This resulted in a reduction of unneeded biopsies of 43% and total number of biopsies of 37% when Proclarix is used, compared to 17% and 16%, respectively by %fPSA only [24]. Here we report the results of the analytical performance of the assays.

Pre-analytical factors including blood sampling and handling can impact the measurement of clinical biomarkers and hamper their reliability and therefore clinical utility. In this instance, since THBS1 is released from platelets during the clotting process, its concentration is affected by the method of blood processing [25]. Further, differences in the measurements of several serum analytes depending on the kind of tubes used for sample preparation were reported [26]. Therefore, we additionally addressed to what extent THBS1 and CTSD levels in serum samples vary depending on blood collection tubes, duration of coagulation, centrifugation conditions and temperature of sample storage.

## Materials and methods

### Specimen collection and sample preparation

**Fig 1:**
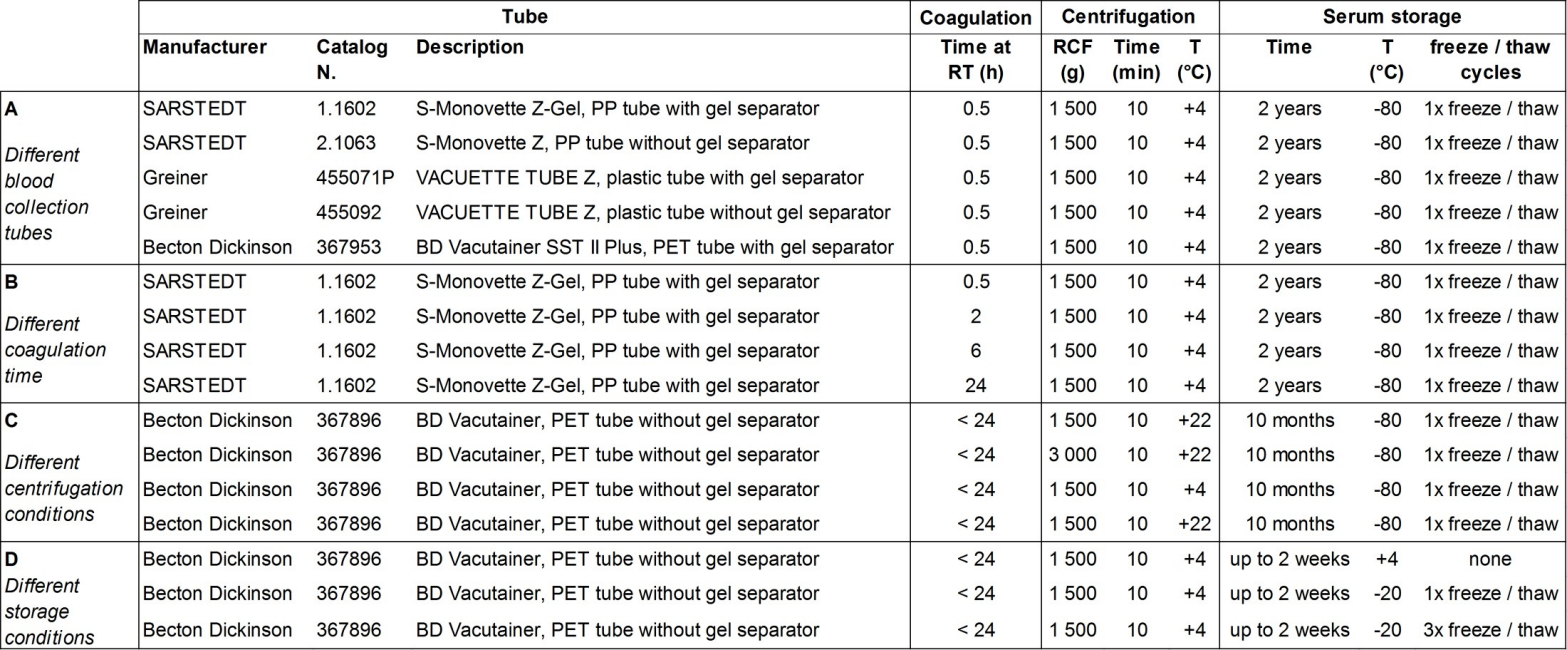
Study design to investigate the impact of pre-analytical factors. Four different sets of *n* matched serum samples were prepared according to the indicated experimental conditions for blood collection (A, *n* = 4), processing (B and C, *n* = 4) and storage (D, *n* = 7).

Blood was obtained from male healthy donors from Blutspende (Schlieren, Switzerland) or provided by Biomex (Heidelberg, Germany). All donors provided written informed consent. Research using such human samples was conducted according to the principles of the Declaration of Helsinki and is approved by Proteomedix’ review committee. As illustrated in Fig 1, for a first series of tests, blood from 4 donors was collected in 5 different tubes with clotting activator and with or without gel separator for serum preparation. Blood was kept at room temperature for 30 min and then centrifuged for 10 min at 1 500 g and 4°C. For a second series of tests, blood from each donor was collected in 4 separate but identical tubes and centrifuged either 0.5, 2, 6 or 24 h after blood draw. For a third and fourth series of tests, blood from each donor was collected in 2 separate but identical tubes and centrifuged either the same day or the following day at 22°C and 1 500 g or 3 000 g, or at 1 500 g and 4°C or 22°C. Samples were analyzed at different time points either without freezing or with one freeze/thaw cycle at -20°C. For the preparation of plasma, blood was collected in S-Monovette CPDA1 tubes, 01.1610.001 SARSTEDT (Newton, United States) and kept at room temperature for 30 min.

### Tumor marker assays

THBS1 and CTSD were measured following the instructions for use of the individual ELISAs (part of the CE-marked product Proclarix, Proteomedix, Switzerland), each comprising plates pre-coated with capture antibody, lyophilized controls (undiluted serum from individual donors) and calibrators (pools of sera from several donors, diluted in lyophilization buffer) to be reconstituted in assay buffer, detection antibody labelled with biotin in a ready-to-use solution, streptavidin-horseradish peroxidase (HRP) and TMB chromogenic substrate solutions. Different assay lots were independently manufactured using different lots of reagents. Since neither reference methods nor international standards for THBS1 and CTSD are available, calibrators were dose-assigned using the newly developed assays and THBS1 purified from human platelets (Athens Research & Technology, Athens, USA) and purified recombinant full-length CTSD (customized production by InVivo BioTech Services, Hennigsdorf, Germany) as reference material (>90% purity by Coomassie staining), with photometrically-determined concentrations. Antibodies are mouse monoclonals previously described [3]. All incubations were performed at 37°C and shaking at 650 rpm in a ThermoMixer C (Eppendorf, Hamburg, Germany). After equilibration at room temperature, pre-coated plates were incubated with biotinylated detection antibody (50 μl for THBS1 or 25 μl for CTSD) and calibrators or pre-diluted (1:1,681 for THBS1 or 1:41 for CTSD) serum samples or controls (50 μl of THBS1 or 100 μl for CTSD) in assay buffer for 1 h. Then plates were washed 5 times with 300 μl wash buffer with a HydroSpeed washer (Tecan, Männedorf, Switzerland) and incubated with 100 μl HRP solution for 30 min. After washing, 100 μl TMB substrate solution was added for color development. After 30 min, 100 μl stop solution was added and absorbance at 450 nm with reference wavelength 620 nm was measured using an Infinite F50 plate reader (Tecan). Calibration curves were generated with Magellan software 7.0 (Tecan) applying a 5-parameter logistics regression and used for determining the diluted sample concentrations. All measurements were performed in duplicate using two independently prepared dilutions. The analyte concentration in serum samples was calculated by multiplying the average concentration of the two measured dilutions by the respective dilution factor. Runs and measurements were valid when values of calibrators and controls and CVs of duplicates were within specified acceptance criteria.

### Analytical performance measurements

Performance characterization was done using sera from healthy donors, which are deemed equivalent to sera from patients in need of a test for the diagnosis of PCa because the levels of THBS1 and CTSD in healthy donors are spread over the whole assay ranges.

Precision was measured based on CLSI guideline EP05 [27] and determined by measuring 16 samples (with measured concentrations of 14–47 ng/ml THBS1 and 6–15 ng/ml CTSD) in duplicate (independent dilutions) by 3 operators in 6 runs over 6 days within the same laboratory. Reproducibility was determined by measuring 15 samples in duplicate in 1 to 6 runs in two laboratories. Due to different measurement conditions, differences between laboratories were considered as possible systematic errors and analysed as biases according to CLSI guideline EP09 [28]. All measurements were repeated with three lots.

Following CLSI guideline EP17 [29], 5 serum samples were measured 4 times in triplicate (total of 60 data points) and parametric approach (assuming normal distribution) was used to determine LoD and LoQ values.

To investigate dilutional linearity and potential hook effect, serial dilutions of 3 serum samples were prepared so that measured concentrations ranged from above the highest to below the lowest calibrator (1:11.2 to 1:20 589 for THBS1 and 1:7.6 to 1:26 for CTSD). Each dilution was measured twice in the same run.

Interfering substances were added to 4 serum samples at concentrations recommended in CLSI guideline EP37, which are typically three times the highest drug concentrations expected during treatment, except when such a high concentration is not achievable [30]: biotin (Sigma 4501) at 2 and 4 mg/ml; bilirubin (B4126 Sigma, St Louis, USA) at 200 and 400 μg/ml; hemoglobin (H7370 Sigma) at 5, 10 and 20 mg/ml; human serum albumin (A8763 Sigma) at 6 and 60 g/l; intralipid (I141 Sigma) at 0.5, 1 and 2%. Hemoglobin and albumin were directly dissolved in the samples. Bilirubin and intralipid were spiked from concentrated solutions (0.1 M solution in sodium-hydroxide and 20% solution, respectively). Interferences by human anti-mouse antibodies (HAMA) or rheumatoid factor (RF) were tested by comparing the measured analyte concentration in 6 sera with elevated HAMA (55–142 ng/ml) and 7 sera with elevated RF (107–523 U/ml) concentrations in the absence or presence of HAMA/RF blocking reagent (85R-1001, Fitzgerald Industries, North Acton, USA).

### Data analyses

Precision was determined applying Variance Component Analysis. Log-scaled measurement data was evaluated using R and the remlVCA function (package ‘VCA’) with random effects ‘sample’, ‘lot’ and ‘day’ (‘day’ nested within ‘lot’). Natural log-scaled data is used to provide estimated variance components. Assuming a constant CV over the investigated concentration range, variance components were pooled over the samples by including the factor ‘sample’ as a trivial random factor in the model on the highest hierarchy level. The related standard deviations are approximate estimates for CV on original units scale. For determination of reproducibility and bias due to measurements in different laboratories, data were analyzed using R and the lmer function (package ‘lme4’) to fit a model including fixed factor ‘laboratory’ (i.e. ‘Laboratory 2’ vs ‘Reference laboratory’), as well as random factors ‘sample’, ‘lot’ and ‘day’, where ‘day’ has been modelled as a nested (within lot and laboratory) effect [31]. The results were pooled over samples and the 90% Confidence Interval (CI) for the fixed effect’s estimates was calculated in terms of asymptotic (‘Wald’) CI using R procedure confint (package ‘lme4’).

Analysis of the influence of pre-analytical factors was performed by normalizing the sample concentrations in the presence of interfering substances to the respective concentrations without interfering substances, or taking the most representative condition as 100% reference. Data were analyzed with Prism V6.07 (GraphPad, La Jolla, USA) and plots reporting the calculated median, 25% to 75% percentile (boxes) and minimum to maximum (whiskers) were generated. Dotted lines at 80% and 120% correspond to predefined limits and provide a reference to discuss results. The statistical analysis of robustness of data was performed using the TOST (two one sided test approach) equivalence testing to determine whether the mean +/- 95% CI is within the predefined 80%-120% limits [32]. Multiple testing was not taken into account. p-values <0.05 indicate equivalence within predefined limits.

## Results

### Calibration curves and analytical sensitivity

To allow measurements of THBS1 and CTSD in diagnostic laboratories, sandwich ELISAs were developed consisting of pre-coated plates and ready to use solutions or lyophilized reagents that are stable for at least 18 months upon refrigeration at 2–8°C. To be compatible with the routine measurements of PSA, the assays were optimized to accurately measure analytes in serum samples.

**Fig 2:**
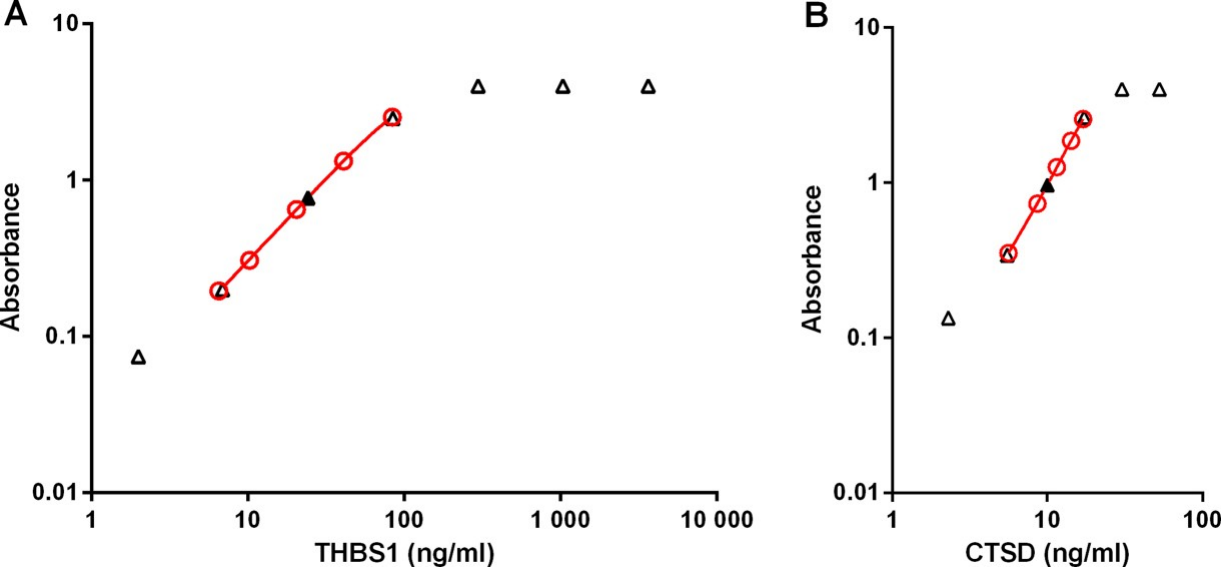
THBS1 and CTSD ELISA calibrator and sample curves. Five calibrators with different levels of THBS1 (A) and CTSD (B) were measured to generate calibration curves (red, open circles) for the determination of the concentration of THBS1 and CTSD in serum samples measured at the recommended dilution (filled triangle). Dose response of one sample representative of 3 measured at different dilutions is plotted using the concentration measured at the recommended dilution and the theoretical concentrations calculated based on the respective dilutions for the other points (open triangles).

**Fig 3:**
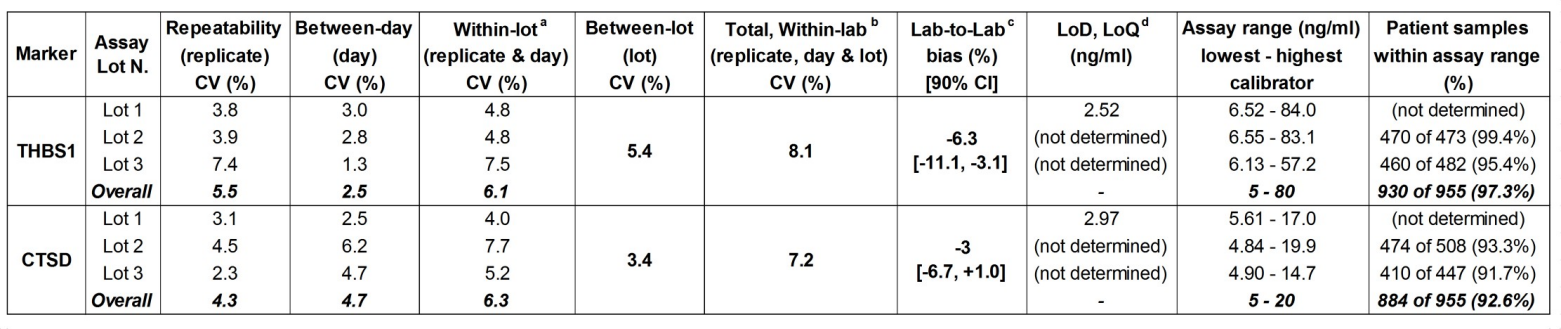
THBS1 and CTSD ELISA characteristics. 16 samples were measured in 6 runs over 6 days within the same laboratory: ^a^ Total including repeatability and between-day variation; ^b^ Total including repeatability, between-day and between-lot variation. ^c^ For the reproducibility, 15 samples were measured in *n* runs in two laboratories with 3 lots (THBS1: Lot 1, *n* = 2; Lot 2 and 3, Ref Lab *n* = 1, Lab 2, *n* = 2. CTSD: Ref Lab *n* = 6, Lab 2, *n* = 2). Measurements in a second laboratory are reported as overall % differences from measurements in reference laboratory based on 3 lots. ^d^ As per CLSI EP17 guideline, since the calculated LoQ is smaller than LoD, the LoD = LoQ.

Five calibrators ranging from approximately (exact concentrations are lot-specific) 5 to 80 ng/ml for THBS1 and from approximately 5 to 20 ng/ml for CTSD are used to generate a calibration curve (Figs 2 and 3).

For the determination of the precision, measurements of the same samples (*n* = 16) were performed with 3 different lots in 6 runs, each on a different day (the source contributing to the reported CV is indicated in parenthesis). Concentrations in a second laboratory (*n* = 15) were measured with 3 different lots in 2 runs. Patient samples were measured only with lot 2 and lot 3 and at the suggested dilution.

To ensure optimal results, extrapolation beyond the lowest and highest calibrators was not allowed and these calibrators were set as the lower and upper limits of quantification. These assay ranges allowed the quantification of >90% of patient serum samples when using the suggested pre-dilutions (Fig 3) [24]. The few samples that fell outside of assay ranges could be quantified by re-measuring them either at a higher or lower dilution. Valid clinical sample measurements (CV between duplicate concentrations ≤15%) were 99.48% for THBS1 and 99.58% for CTSD.

### Analytical specificity

The antibody pairs were specific for THBS1 or CTSD as determined by signal below LoQ when testing binding to related proteins THBS2, THBS3, THBS4, THBS5, or CTSE, pepsin A-5, renin, respectively (S1 Table).

### Linearity and hook effect

Serial dilutions of serum samples generated a curve parallel to the calibration curve and showed that at high concentrations of analyte the assays are in saturation. No hook effect was observed at concentrations as high as 4,872 ng/ml for THBS1 (60 times higher than the highest calibrator) and 61.2 ng/ml for CTSD (3.6 times higher than the highest calibrator) (Fig 2).

### Precision

**Fig 4:**
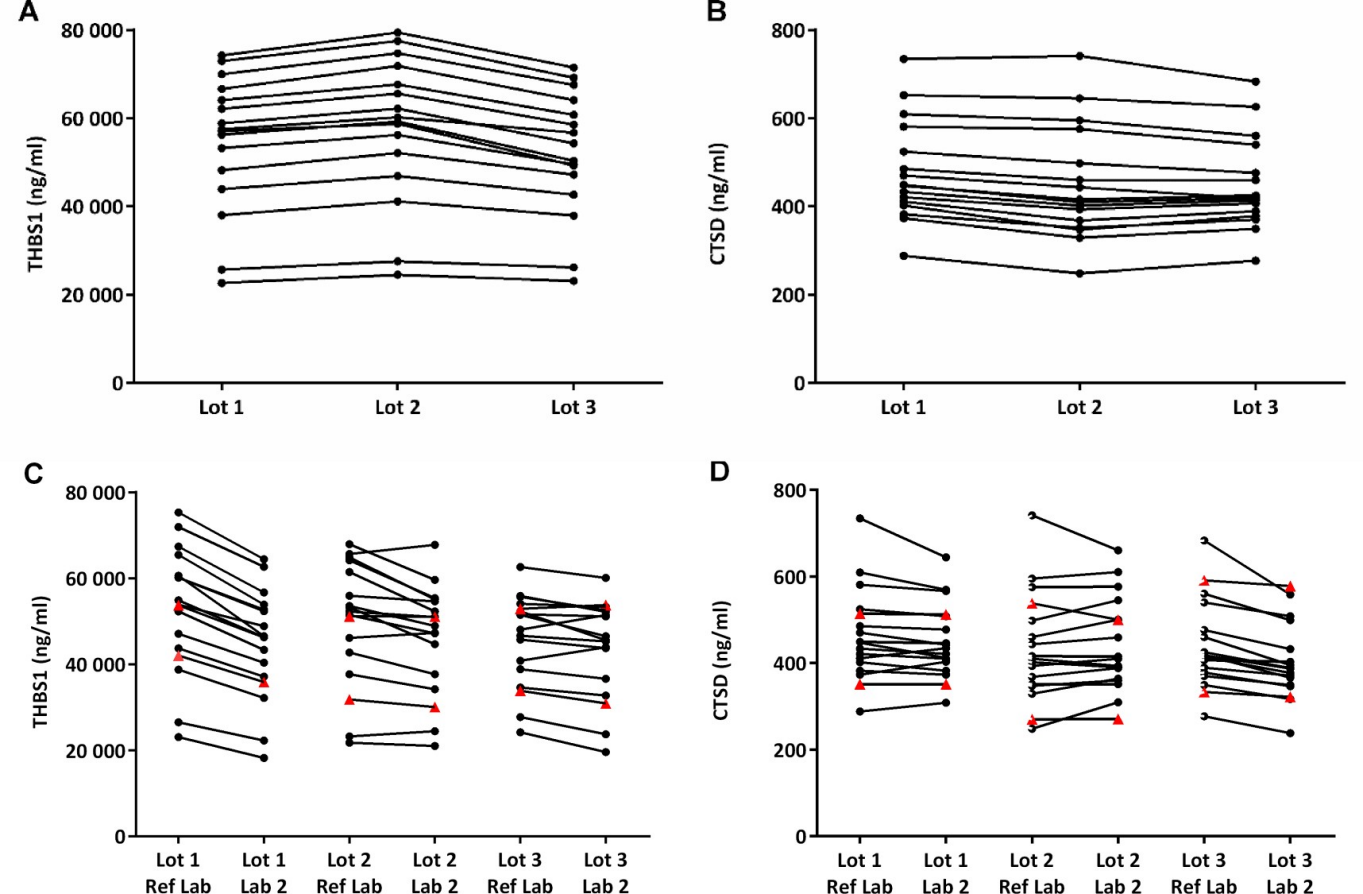
Concentration determination with different lots and by different laboratories. (A and B) Reported are the averages of 6 determinations in reference laboratory (Ref Lab). (C and D) Reproducibility was tested by measuring samples in duplicate in *n* runs in reference laboratory (Ref Lab) and in independent laboratory (Lab 2). Reported are the concentration determinations of the averages of *n* runs (THBS1: Lot 1, *n* = 2; Lot 2 and 3, Ref Lab *n* = 1, Lab 2, *n* = 2. CTSD: always *n* = 2) of samples and controls. Each line represents one sample. Filled red triangles represent concentrations of controls.

Intra-CVs of replicate measurements (repeatability) were 5.5% for THBS1 and 4.3% for CTSD, whereas the within-lot CVs (including variation of different replicates and days, but excluding variation of lots and laboratories) were 6.1% for THBS1 and 6.3% for CTSD (Fig 3). To determine the spread of the analyte concentrations depending on the lot used for the measurements, a panel of 16 serum samples was measured with 3 lots. THBS1 concentrations determined with lot 2 and lot 3 were respectively slightly higher or lower compared to quantification with lot 1, but well within the 80%-120% predefined limits of variation (Figs 3 and 4A).

For CTSD, concentrations measured with lot 2 and lot 3 were similar and with lot 1 slightly higher. The total within-laboratory inter-CVs (including variation of different replicates, days and lots) were 8.1% for THBS1 and 7.2% for CTSD. When the same samples were measured in two independent laboratories (reproducibility), concentrations for CTSD were similar for lot 1 and lot 2 and slightly shifted to lower values with lot 3 (Figs 3 and 4D). For THBS1, concentrations measured in a second laboratory were similar for lot 2 and 3, but for lot 1 they were shifted to slightly lower values compared to measurements performed in reference laboratory, with an estimated overall bias of -6.3% (Figs 3 and 4C). Similar patterns were observed also for the controls provided with the kits (Fig 4C and 4D) indicating that potential biases can be readily detected. Despite these differences, these results show that independent of the lot used and of the laboratory performing the assays, measurements differ by less than 20% (Fig 3).

#### Interferences

**Fig 5:**
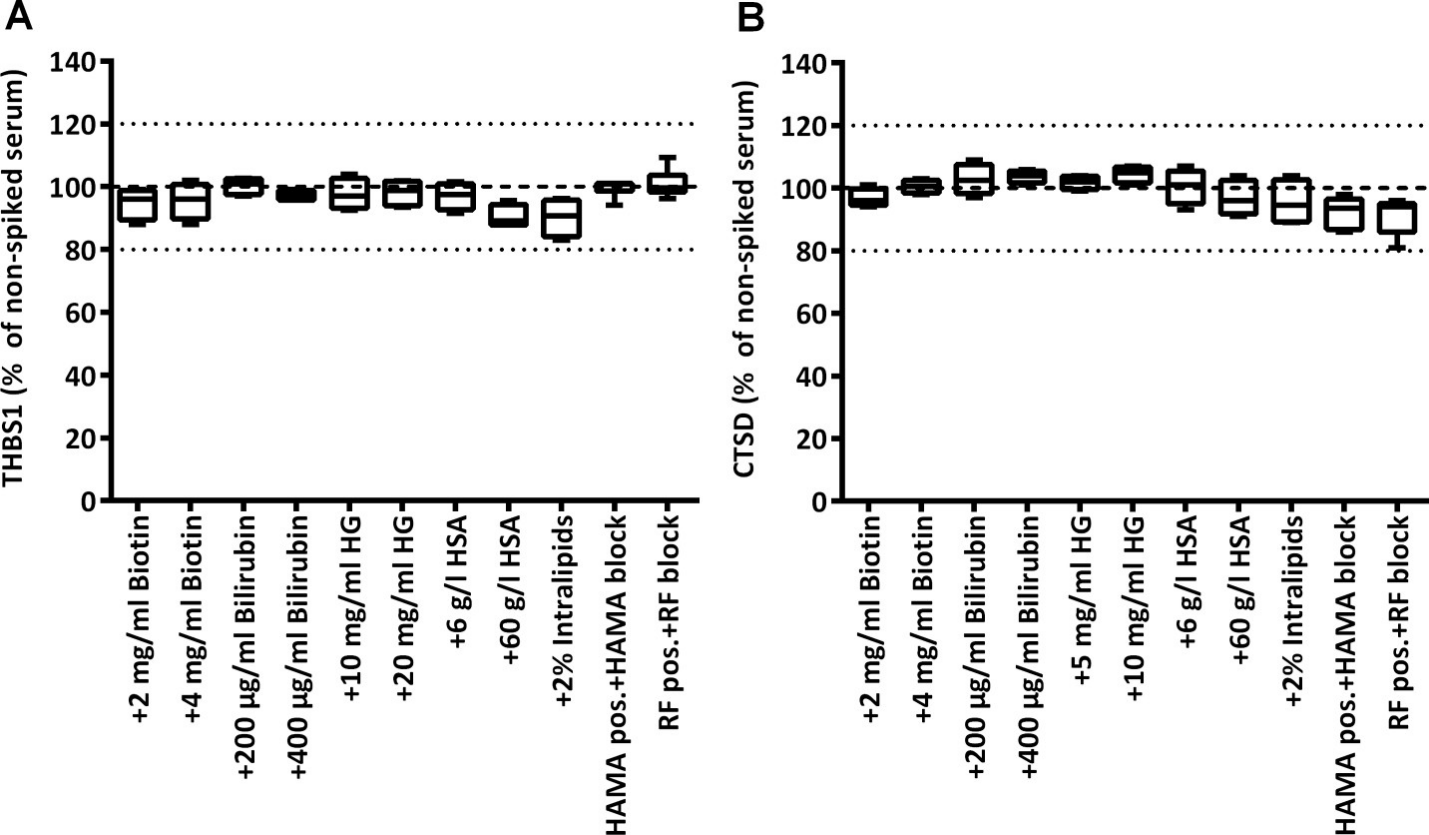
Impact of common interfering substances on measured marker levels. THBS1 and CTSD were measured in serum samples before and after spiking of biotin, bilirubin, hemoglobin (HG), human serum albumin (HSA), or intralipids (*n* = 4). Sera with high titer of HAMA (*n* = 6) or RF (*n* = 7) were measured with or without addition of HAMA/RF blocking reagent. Plots show the median, the 25 to 75% percentile (boxes) and minimum to maximum (whiskers) of relative serum levels of THBS1 (A) and CTSD (B) in samples with potentially interfering substances, taking as 100% reference the levels in the respective samples non-spiked/spiked with buffer only/without addition of blocker. TOST equivalence testing resulted in p-values <0.01 for all conditions except +2% intralipids (THBS1, p = 0.0217; CTSD, p = 0.0117).

**Fig 6:**
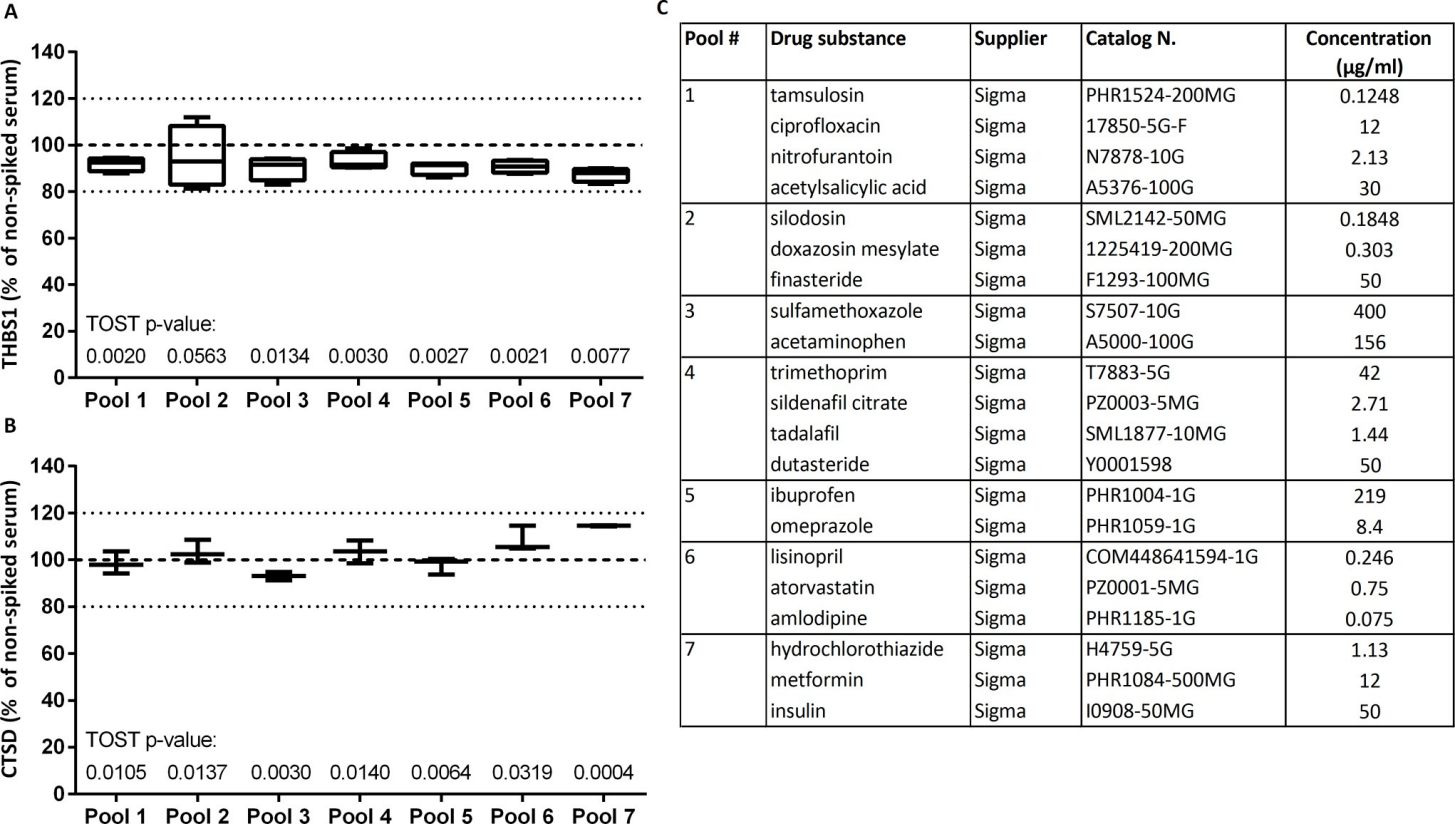
Impact of drug substances on measured marker levels. THBS1 and CTSD were measured in serum samples before and after spiking 7 pools of drug substances. Plots show the median, the 25 to 75% percentile (boxes) and minimum to maximum (whiskers) of relative serum levels of THBS1 (*n* = 4) (A) and CTSD (*n* = 3) (B) in samples with potentially interfering drugs, taking as 100% reference the levels in the respective non-spiked serum (carrier buffer only). Reported are p-values for TOST equivalence testing. (C) Spiked concentration and compositions of pools.

To test robustness of measurements, we checked whether quantification of the biomarkers is affected by the presence of potentially interfering common substances, at concentrations that are typically three times the highest concentration expected during treatment. The percent difference from concentrations in sera without interference substances or without blocking reagent was below 20% for all samples in both assays (Fig 5). The same was true for samples spiked with several drug substances (Fig 6). The TOST test showed that the measured concentrations in the presence and absence of potentially interfering material were equivalent (except one, all p-values <0.05).

### Variation depending on serum sample preparation

**Fig 7:**
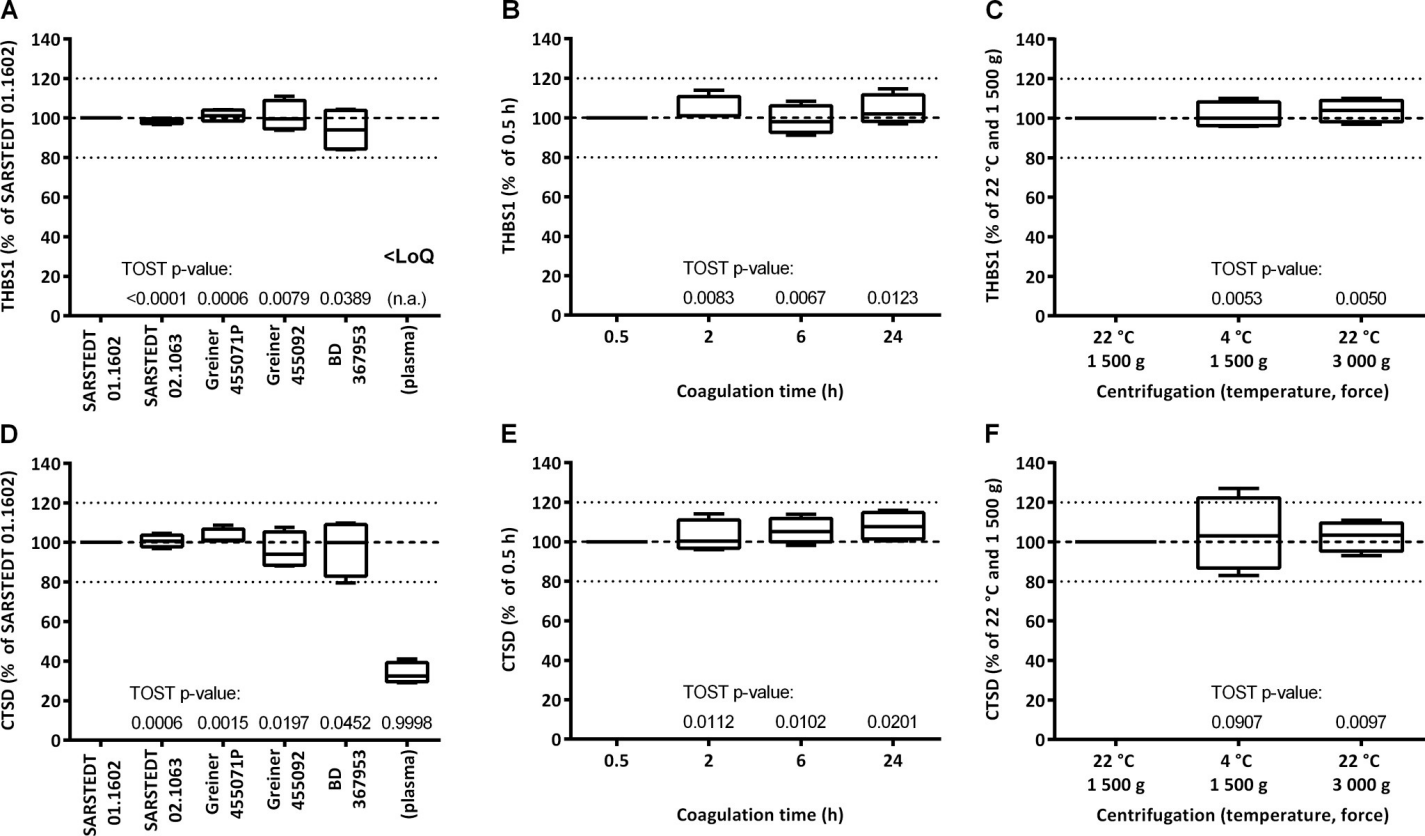
Relative marker levels in serum depending on sample preparation procedure. Whole blood from matched donors was collected in different tubes (A and D), let stand at RT for different time before centrifugation (B and E), and centrifuged at different temperature and force for the separation of serum (C and F). Plots show the median, the 25 to 75% percentile (boxes) and minimum to maximum (whiskers) of relative serum levels of THBS1 (A, B, C) and CTSD (D, E, F), taking as 100% reference the respective concentrations in serum prepared with Sarstedt 01.1602 tubes (*n* = 4) (A and D), of the 0.5 h samples (*n* = 4) (B and E), and samples centrifuged at 1 500 g and 22°C (*n* = 4) (C and F). Reported are p-values for TOST equivalence testing (n.a., not applicable).

Pre-analytical factors possibly affecting routine measurements of the samples were assessed (Fig 1). One main source of variation is the tube used for collection of whole blood and preparation of serum. Tubes from several manufacturers all containing clot activator but differing in material and presence of gel separator were tested. Quantification of THBS1 and CTSD in matched serum samples derived from whole blood of the same donor collected in different tubes showed that there is small variation and no clear trend (Fig 7A and 7D). In particular, we determined that measured relative concentrations fall between 84% and 111% for THBS1 and between 80% and 110% for CTSD. Of note, concentrations of THBS1 and CTSD measured in plasma are much lower. Plasma THBS1 is below the limit of quantification and plasma CTSD levels are <30% of serum levels (Fig 7A and 7D).

Once collected in tubes, whole blood is left at room temperature to allow clotting whereby the optimal time recommended by manufacturers is 30 min. However, in a realistic routine setting in which blood is withdrawn by the physician and sent to laboratories for the measurement, incubation time before centrifugation for the separation of serum can be of several hours. Further, centrifugation conditions might vary in terms of centrifugal force and temperature. Quantification of THBS1 and CTSD in matched serum samples derived from whole blood stored at room temperature for 0.5, 2, 6 and 24 h showed that there is some variation, but this is relatively small (Fig 7B and 7E). We determined that measured relative concentrations fall between 91% and 115% for THBS1 and between 96% and 116% for CTSD. When testing different centrifugation conditions, results varied depending on the sample, but altogether there was no difference (Fig 7C and 7F).

### Variation depending on serum sample storage

**Fig 8:**
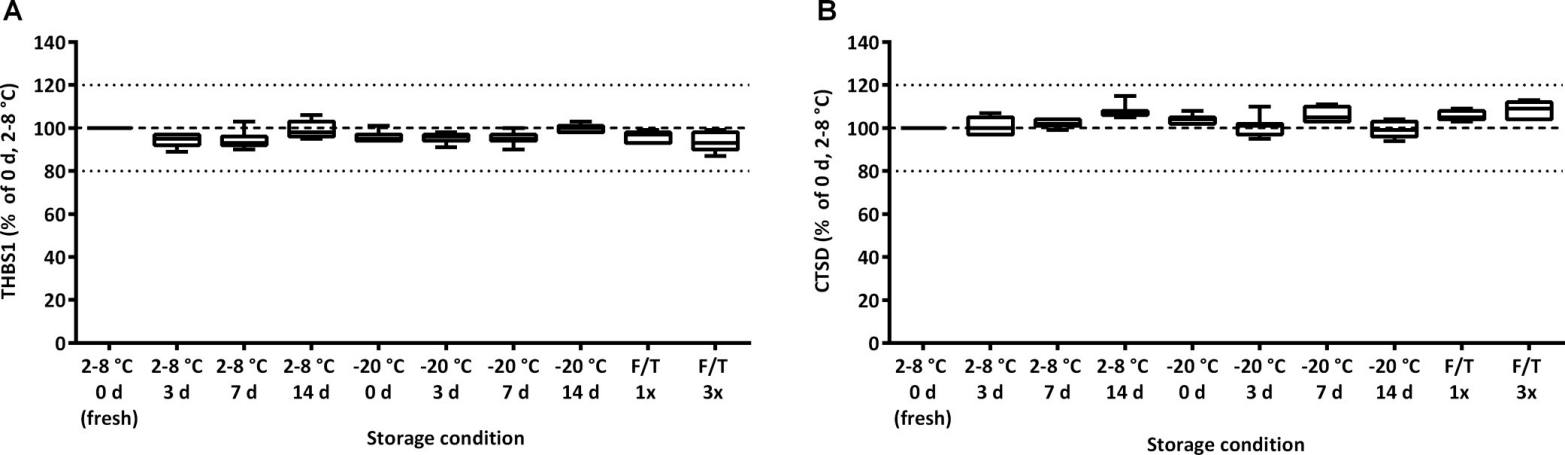
Stability of THBS1 and CTSD upon different storage temperature. After separation, aliquots of serum samples were stored refrigerated (2–8°C) or frozen (-20°C) for 1 d, 7 d and 14 d. At each time point one aliquot per condition was taken to measure THBS1 (A) and CTSD (B) levels. One additional aliquot was subjected to 3 freeze/thawing cycles over 1 week. Plots show the median, the 25 to 75% percentile (boxes) and minimum to maximum (whiskers) of relative serum levels, taking as 100% reference the respective concentrations of samples measured immediately after centrifugation (2–8°C 0 d, fresh) (*n* = 7). TOST equivalence testing: p-value <0.0001.

To ensure accurate results, stability of analytes in undiluted serum under different storage conditions is critical. Once serum is separated, samples are usually stored at 2–8°C until they are measured. If longer storage is required, samples are frozen (-20°C) and thawed just prior to analysis. Measurements of matched serum samples analyzed immediately after separation (fresh samples, with no freeze/thaw cycle), stored at 2–8°C for 3 days, 1 or 2 weeks, or stored at -20°C and then thawed, showed that there is no difference in quantification of THBS1 and CTSD (Fig 8).

## Discussion

The high rate of false positives resulting from the use of the PSA test for screening for PCa highlights the need to develop new tests for improving the diagnosis especially for patients in the diagnostic grey zone with elevated PSA, negative DRE and enlarged prostate. THBS1 and CTSD play a role in several types of tumors and we found that they can aid the diagnosis of high-grade PCa [4, 6, 7, 19]. Therefore, we developed robust assays to quantify these markers in serum samples and included them in a commercial IVD test (Proclarix) [24]. This study reports the analytical performance of the individual THBS1 and CTSD ELISAs.

We showed that the assays are suitable for the determination of THBS1 and CTSD concentrations in more than 90% of patient serum samples in the first run (out-of-range samples require retesting at a different dilution), with a high precision (total within-laboratory CV 8.1% for THBS1 and 7.2% for CTSD).

Our THBS1 and CTSD ELISAs are two-site immunoassays in which capture and detection antibodies are simultaneously exposed to the analyte. High-dose hook effect has been shown for several analytes (PSA, growth hormone, ferritin) [33–35] and is dependent on the sample to antibody ratio, where in the presence of high concentrations of analyte, binding of the labelled antibody-bound analyte may be decreased rather than increased (due to competitive binding of free analyte), resulting in erroneous results. When sera were tested at lower dilutions, the signal for both assays increased and reached saturation, never dropping lower than the signal of the highest calibrator. These results show that there is no hook effect and ensure that determined concentrations are correct. Further, the relative difference between measurements with different lots or performed by different laboratories was <15%. This low extent of variation shows that overall assays are reliable for the accurate quantification of the markers according to their intended use. In addition, we expect that conductance of assays on fully-automated workstations (e.g. DSX from Dynex Technologies) will further improve assay performance. Importantly, controls provided with the kit for quality purposes allow detection of measurement errors. By comparing measured concentrations with provided acceptable ranges, they determine whether performance is acceptable and measurements are valid, thereby limiting possible errors. Should their measured concentrations fall outside of the provided ranges, all measurements from the same run are invalid and should be repeated.

Substances that alter the binding of the analyte to the antibodies used in the assay, and therefore its measurable concentration, are known as immunoassay interference substances. Interferences lead to artefactual laboratory results that may cause clinical misinterpretation, incorrect diagnosis and course of treatment suggested by the physician [33]. A well-known interference is caused by anti-animal antibodies (particularly HAMA), which specifically bind to capture and detection reagents. Though varying widely, their prevalence in animal workers and patients on monoclonal antibody therapy is up to 80% [36]. Our results show that THBS1 and CTSD are accurately measured in the presence of potentially interfering substances biotin, bilirubin, hemoglobin, albumin, intralipid, HAMA or RF, as well as in the presence of several drug substances common for the intended use population. This largely reduces the adverse possibility that results are dependent on the physiological conditions of the patients.

THBS1 is an anti-angiogenic factor that is abundantly stored in platelets and is released during coagulation due to platelet activation. Therefore, compared to plasma levels THBS1 serum levels are very high [37]. Notably, it has been shown that suboptimal blood processing can result in inadvertent partial platelet activation and fluctuating THBS1 concentrations in plasma with differences up to 10-fold, making plasma samples not optimal for reliable measurements [25]. Similarly to VEGF, the difference in the role of THBS1 in plasma compared to THBS1 stored in platelets is unclear, especially in relation to cancer [18]. To be compatible with the routine measurements of PSA, which are performed in serum, we decided to evaluate serum THBS1 as biomarker for PCa. Though not directly suggesting a specific role of platelet-stored THBS1 in PCa progression, the results of our clinical studies measuring THBS1 in the serum of patients belonging to two different cohorts validate the value of serum THBS1 in aiding the diagnosis of PCa [3, 4, 23, 24]. Therefore, our CE-marked Proclarix test was developed to measure serum and not plasma THBS1. On the other hand, serum samples might suffer from similar limitations depending on blood sampling and processing procedures. Such pre-analytical factors have been shown to influence the quantification of analytes in clinical chemistry as well as in immunoassays, for example for the quantification of free PSA [26, 38, 39]. Our results show that serum levels of THBS1 and CTSD are largely unaffected by pre-analytical factors, with measured concentrations relative to reference conditions between 80% and 120%. Additionally, we found that both biomarkers are stable in serum samples stored at 2–8°C for up to 2 weeks and that results are unaffected by freezing at -20°C and subsequent thawing before measurements. This provides high flexibility to the analytical laboratories for storage of samples and planning measurements of multiple samples prepared in different days. Based on all these results we conclude that the assays are robust from a sampling and storage perspective to be easily used in clinical and laboratory routine practice.

In conclusion, the assays are suitable for quantifying THBS1 and CTSD in human serum of patients with prostate disease and their analytical performance is good. Measurements are largely unaffected by pre-analytical procedures that reflect the routine management of samples in the clinical practice. Assay characteristics, format and CE-mark, make our Proclarix test suitable for testing in analytical laboratories in a decentralized fashion, delivering reliable results that can be used to aid the diagnosis of high-grade PCa.

## Supporting information

S1 Table Specificity of THBS1 and CTSD ELISA. Endogenous human serum proteins with potential for cross-reactivity with THBS1 and CTSD were spiked at 10 μg/ml in dilution buffer (physiologic serum concentration ranges are <100 ng/ml for all, except THBS5, which can be as hight as 10 μg/ml and measured at the suggested dilution with the respective ELISA.
(TIF)

## References

[1] Hoffman RM. Clinical practice. Screening for prostate cancer. N Engl J Med. 2011;365(21):2013–9. Epub 2011/10/28. 10.1056/NEJMcp1103642 . 22029754

[2] Cima I, Schiess R, Wild P, Kaelin M, Schuffler P, Lange V, et al Cancer genetics-guided discovery of serum biomarker signatures for diagnosis and prognosis of prostate cancer. Proc Natl Acad Sci U S A. 2011;108(8):3342–7. Epub 2011/02/09. 10.1073/pnas.1013699108 21300890 PMC3044355

[3] Endt K, Goepfert J, Omlin A, Athanasiou A, Tennstedt P, Guenther A, et al Development and clinical testing of individual immunoassays for the quantification of serum glycoproteins to diagnose prostate cancer. PLoS One. 2017;12(8):e0181557 Epub 2017/08/03. 10.1371/journal.pone.0181557 28767721 PMC5540289

[4] Steuber T, Tennstedt P, Macagno A, Athanasiou A, Wittig A, Huber R, et al Thrombospondin 1 and cathepsin D improve prostate cancer diagnosis by avoiding potentially unnecessary prostate biopsies. BJU Int. 2018 Epub 2018/09/15. 10.1111/bju.14540 . 30216634 PMC7379977

[5] Campbell SC, Volpert OV, Ivanovich M, Bouck NP. Molecular mediators of angiogenesis in bladder cancer. Cancer Res. 1998;58(6):1298–304. Epub 1998/03/27. . 9515819

[6] Kazerounian S, Yee KO, Lawler J. Thrombospondins in cancer. Cell Mol Life Sci. 2008;65(5):700–12. Epub 2008/01/15. 10.1007/s00018-007-7486-z 18193162 PMC2752021

[7] Roberts DD. Regulation of tumor growth and metastasis by thrombospondin-1. FASEB J. 1996;10(10):1183–91. Epub 1996/08/01. . 8751720

[8] Shafer MW, Mangold L, Partin AW, Haab BB. Antibody array profiling reveals serum TSP-1 as a marker to distinguish benign from malignant prostatic disease. Prostate. 2007;67(3):255–67. Epub 2006/12/29. 10.1002/pros.20514 . 17192876

[9] Wang TN, Qian X, Granick MS, Solomon MP, Rothman VL, Berger DH, et al Thrombospondin-1 (TSP-1) promotes the invasive properties of human breast cancer. J Surg Res. 1996;63(1):39–43. Epub 1996/06/01. 10.1006/jsre.1996.0219 . 8661169

[10] Yamaguchi M, Sugio K, Ondo K, Yano T, Sugimachi K. Reduced expression of thrombospondin-1 correlates with a poor prognosis in patients with non-small cell lung cancer. Lung Cancer. 2002;36(2):143–50. Epub 2002/04/17. 10.1016/s0169-5002(01)00470-6 . 11955648

[11] Zabrenetzky V, Harris CC, Steeg PS, Roberts DD. Expression of the extracellular matrix molecule thrombospondin inversely correlates with malignant progression in melanoma, lung and breast carcinoma cell lines. Int J Cancer. 1994;59(2):191–5. Epub 1994/10/15. 10.1002/ijc.2910590209 . 7927918

[12] Doll JA, Reiher FK, Crawford SE, Pins MR, Campbell SC, Bouck NP. Thrombospondin-1, vascular endothelial growth factor and fibroblast growth factor-2 are key functional regulators of angiogenesis in the prostate. Prostate. 2001;49(4):293–305. Epub 2001/12/18. 10.1002/pros.10025 . 11746276

[13] Lawler J. Thrombospondin-1 as an endogenous inhibitor of angiogenesis and tumor growth. J Cell Mol Med. 2002;6(1):1–12. Epub 2002/05/11. 10.1111/j.1582-4934.2002.tb00307.x . 12003665 PMC6740251

[14] Miyata Y, Sakai H. Thrombospondin-1 in urological cancer: pathological role, clinical significance, and therapeutic prospects. Int J Mol Sci. 2013;14(6):12249–72. Epub 2013/06/12. 10.3390/ijms140612249 23749112 PMC3709784

[15] Ren B, Yee KO, Lawler J, Khosravi-Far R. Regulation of tumor angiogenesis by thrombospondin-1. Biochim Biophys Acta. 2006;1765(2):178–88. Epub 2006/01/13. 10.1016/j.bbcan.2005.11.002 . 16406676

[16] Russo G, Mischi M, Scheepens W, De la Rosette JJ, Wijkstra H. Angiogenesis in prostate cancer: onset, progression and imaging. BJU Int. 2012;110(11 Pt C):E794–808. Epub 2012/09/11. 10.1111/j.1464-410X.2012.11444.x . 22958524

[17] Vallbo C, Wang W, Damber JE. The expression of thrombospondin-1 in benign prostatic hyperplasia and prostatic intraepithelial neoplasia is decreased in prostate cancer. BJU Int. 2004;93(9):1339–43. Epub 2004/06/08. 10.1111/j.1464-410x.2004.04818.x . 15180634

[18] Gonzalez FJ, Rueda A, Sevilla I, Alonso L, Villarreal V, Torres E, et al Shift in the balance between circulating thrombospondin-1 and vascular endothelial growth factor in cancer patients: relationship to platelet alpha-granule content and primary activation. Int J Biol Markers. 2004;19(3):221–8. Epub 2004/10/27. . 28207087 10.5301/JBM.2008.1959

[19] Benes P, Vetvicka V, Fusek M. Cathepsin D—many functions of one aspartic protease. Crit Rev Oncol Hematol. 2008;68(1):12–28. Epub 2008/04/09. 10.1016/j.critrevonc.2008.02.008 18396408 PMC2635020

[20] El Melegy NT, Aboulella HA, Abul-Fadl AM, Mohamed NA. Potential biomarkers for differentiation of benign prostatic hyperplasia and prostate cancer. Br J Biomed Sci. 2010;67(3):109–12. Epub 2010/10/27. 10.1080/09674845.2010.11730306 . 20973404

[21] Leto G, Tumminello FM, Crescimanno M, Flandina C, Gebbia N. Cathepsin D expression levels in nongynecological solid tumors: clinical and therapeutic implications. Clin Exp Metastasis. 2004;21(2):91–106. Epub 2004/06/01. 10.1023/b:clin.0000024740.44602.b7 . 15168727

[22] Vetvicka V. Procathepsin D in cancer development. Journal of Cancer Therapeutics and Research. 2012;1(1). 10.7243/2049-7962-1-22

[23] Klocker H, Steiner E, Horninger W, Thomas S, Tennstedt P, Macagno A, et al Thrombospondin 1 and cathepsin D improve the detection of high-grade prostate cancer and reduce the number of unnecessary prostate biopsies. European Urology Supplements. 2018;17(2):e544. Epub 2018/03/02.

[24] Klocker H, Golding B, Weber S, Steiner E, Tennstedt P, Keller T, et al Development and validation of a novel multivariate risk score to guide biopsy decision for the diagnosis of clinically significant prostate cancer. BJUI COMPASS. 2020 10.1002/bco2.8. PMC8988838 35474911

[25] Starlinger P, Alidzanovic L, Schauer D, Brugger P, Sommerfeldt S, Kuehrer I, et al Platelet-stored angiogenesis factors: clinical monitoring is prone to artifacts. Dis Markers. 2011;31(2):55–65. Epub 2011/09/08. 10.3233/DMA-2011-0798 21896999 PMC3826483

[26] Bowen RA, Remaley AT. Interferences from blood collection tube components on clinical chemistry assays. Biochem Med (Zagreb). 2014;24(1):31–44. Epub 2014/03/15. 10.11613/BM.2014.006 24627713 PMC3936985

[27] CLSI guideline EP05 Evaluation of Precision of Quantitative Measurement Procedures, 3rd Edition Clinical and Laboratory Standards Institute 2014;(September 17, 2014). Epub Third.

[28] CLSI guideline EP09 Measurement Procedure Comparison and Bias Estimation Using Patient Samples; Approved Guideline, 3rd Edition Clinical and Laboratory Standards Institute 2018;(June 20, 2018). Epub Third.

[29] CLSI guideline EP17 Evaluation of Detection Capability for Clinical Laboratory Measurement Procedures, 2nd Edition Clinical and Laboratory Standards Institute 2012;(18 Jun 2012.).

[30] CLSI guideline EP37 Supplemental Tables for Interference Testing in Clinical Chemistry, 1st Edition Clinical and Laboratory Standards Institute 2018;(18 Dec 2018.).

[31] Pankaj K. Choudhary HNN. Measuring Agreement: Models, Methods, and Applications: Wiley; 2017.

[32] Schuirmann DJ. A comparison of the two one-sided tests procedure and the power approach for assessing the equivalence of average bioavailability. J Pharmacokinet Biopharm. 1987;15(6):657–80. Epub 1987/12/01. 10.1007/BF01068419 . 3450848

[33] Selby C. Interference in immunoassay. Ann Clin Biochem. 1999;36 (Pt 6):704–21. Epub 1999/12/10. 10.1177/000456329903600603 . 10586307

[34] Charrie A, Charriere G, Guerrier A. Hook effect in immunometric assays for prostate-specific antigen. Clin Chem. 1995;41(3):480–1. Epub 1995/03/01. . 7533675

[35] Fernando SA, Wilson GS. Studies of the 'hook' effect in the one-step sandwich immunoassay. J Immunol Methods. 1992;151(1–2):47–66. Epub 1992/07/06. 10.1016/0022-1759(92)90104-2 . 1378475

[36] Kricka LJ. Human anti-animal antibody interferences in immunological assays. Clin Chem. 1999;45(7):942–56. Epub 1999/07/01. . 10388468

[37] Barclay JL, Keshvari S, Whitehead JP, Inder WJ. Development of an enzyme-linked immunosorbent assay for thrombospondin-1 and comparison of human plasma and serum concentrations. Ann Clin Biochem. 2016;53(Pt 5):606–10. Epub 2016/01/10. 10.1177/0004563216628891 . 26748102

[38] Dittadi R, Fabricio ASC, Rainato G, Peroni E, Di Tonno F, Vezzu B, et al Preanalytical stability of [–2]proPSA in whole blood stored at room temperature before separation of serum and plasma: implications to Phi determination. Clin Chem Lab Med. 2019;57(4):521–31. Epub 2018/09/16. 10.1515/cclm-2018-0596 . 30218601

[39] Zhao X, Qureshi F, Eastman PS, Manning WC, Alexander C, Robinson WH, et al Pre-analytical effects of blood sampling and handling in quantitative immunoassays for rheumatoid arthritis. J Immunol Methods. 2012;378(1–2):72–80. Epub 2012/03/01. 10.1016/j.jim.2012.02.007 22366959 PMC3404505

